# Investigating Risk Factors for Delayed Melanoma Recurrence Beyond 5 Years Post‐Primary Tumor Diagnosis

**DOI:** 10.1002/cam4.72389

**Published:** 2026-10-28

**Authors:** Kaspar Itin, Rebecca Lapides, Soumya Paria, Babak Saravi, Alina. M. Torrez, Lara Valeska Maul, Michael Wang‐Evers, Alexander A. Navarini, Dieter Manstein, Beata Kun, Leticia Szadai, Judit Olàh, Eszter Baltas, Rolland Gyulai, Peter Hollo, Grzegorz Sarek, Istvan B. Németh, Kaustubh Adhikari, Elisabeth Roider

**Affiliations:** ^1^ Department of Dermatology University Hospital of Basel Basel Switzerland; ^2^ Robert Larner, M.D. College of Medicine at the University of Vermont Burlington Vermont USA; ^3^ Cutaneous Biology Research Center, Department of Dermatology Massachusetts General Hospital, Harvard Medical School Charlestown Massachusetts USA; ^4^ Faculty of Science, Technology, Engineering & Mathematics The Open University Milton Keynes UK; ^5^ Department of Orthopedics and Trauma Surgery, Medical Center, Faculty of Medicine University of Freiburg Freiburg Germany; ^6^ Department of Dermatology University Hospital Zurich Zurich Switzerland; ^7^ Faculty of Medicine University of Zurich Zurich Switzerland; ^8^ Department of Dermatology, Venereology and Dermatooncology Semmelweis University Budapest Hungary; ^9^ Department of Dermatology and Allergology Szent‐Györgyi Albert Medical School, University of Szeged Szeged Hungary; ^10^ School of Mathematics and Statistics, Faculty of Science, Technology, Engineering and Mathematics The Open University Milton Keynes UK; ^11^ Department of Genetics, Evolution and Environment, and UCL Genetics Institute University College London London UK

**Keywords:** BRAF, late recurrence, melanoma, patient management, risk factors

## Abstract

**Background:**

Patients undergoing treatment for cutaneous melanoma are generally considered cured after a period of 5 years, since most recurrences manifest within three to 5 years. However, some patients can present with a delayed recurrence of melanoma beyond a 10‐year timeframe.

**Objective:**

To identify clinicopathologic factors associated with survival in patients with cutaneous melanoma who developed metastatic recurrence at least 5 years after primary diagnosis.

**Methods:**

We conducted a retrospective single‐center cohort study of 30 patients with cutaneous melanoma developing metastatic recurrence at least 5 years post‐diagnosis. The cohort contained 18 women and 12 men (mean age 50.2; range 25–76). Variables included age, sex, BRAF status, site, melanoma subtype, Breslow thickness, ulceration, Clark level, regression, and recurrence localization. Overall survival (OS), progression‐free survival (PFS), and post‐recurrence survival (PRS) were evaluated via Cox proportional hazards models.

**Results:**

Mean OS, PFS, and PRS were 168.7, 130.4, and 43.7 months, respectively. Eight patients (27%) died. Tumors averaged 2.61 mm Breslow thickness; 50.0% were BRAF‐mutant, 53.3% ulcerated, and 50.0% truncal. Recurrences were locoregional (cutaneous, subcutaneous, in‐transit, nodal) in eight (26.7%) and distant in 21 (70.0%); one lacked data. Female sex predicted better univariate OS (HR 0.055, 95% CI 0.01–0.47; *p* = 0.008) and multivariate PFS (HR 0.364, 95% CI 0.14–0.93; *p* = 0.035). Increasing age predicted shorter multivariate PFS (HR 1.072, 95% CI 1.03–1.12; *p* = 0.001). Histologic regression (HR 3.557, 95% CI 1.04–12.17; *p* = 0.043) and BRAF status (HR 0.413, 95% CI 0.18–0.94; *p* = 0.034) independently impacted PFS. Kaplan–Meier showed longer OS and PFS in women, and longer PFS in patients younger than 50 years.

**Conclusions:**

Delayed recurrence can manifest over a decade post‐diagnosis. Female sex and younger age correlate with favorable survival. These findings underscore the necessity of continued clinical vigilance and patient counseling beyond 10 years, although larger cohorts are needed to validate late‐recurrence risk stratification.

## 1 Introduction

Cutaneous melanoma is an aggressive skin cancer that requires prompt treatment and long‐term surveillance to reduce the risk of disease progression. According to current EADV guidelines, patients with stage 0‐II melanoma are generally followed every 3–12 months during the first 5 years after complete excision and annually thereafter. This follow‐up schedule reflects the observation that most recurrences occur within the first 5 years, particularly during the first 3 years after the initial diagnosis [1]. Despite these established guidelines, up to 6.9% of patients experience “late recurrence,” defined as recurrence more than 10 years after the initial diagnosis [2, 3, 4, 5, 6, 7, 8, 9, 10].

Research on late recurrence remains fragmented and limited, as much of the available evidence is derived from case reports, small series, or retrospective cohorts. Differences in study design, cohort composition, and definitions of late recurrence further complicate the development of standardized follow‐up recommendations [6]. Larger cohort studies have suggested that late melanoma recurrence is more common in younger patients and in those with thinner primary tumors [4, 6, 7]. However, these associations have not been observed consistently across studies, and some reports have failed to identify reproducible clinicopathologic predictors of late recurrence [7, 11, 12]. In addition, conventional pathologic descriptors of the primary melanoma remain clinically relevant when evaluating late relapse. These include Breslow thickness, ulceration, Clark level, anatomic primary site, and anatomic pattern of relapse, all of which are established components of melanoma prognostication and may influence the biology and timing of recurrence. Beyond these intrinsic histopathologic features of the primary tumor, host systemic factors play a critical role in mediating the latency and timing of disease recurrence. A particularly complex variable is the role of immunocompetence in melanoma recurrence, with factors such as age and sex potentially influencing immune surveillance and, therefore, the probability of late recurrence [11]. When this protective immune surveillance eventually wanes, it allows residual disease to persist undetected.

Prolonged immune suppression raises critical questions about tumor cell behavior during clinical invisibility, prompting the development of several biological models to explain this extended period of undetectable disease. Some hypotheses propose that melanoma dormancy involves a complex interplay among subclinical proliferation, active immune suppression, and cellular quiescence, which together dictate post‐reactivation survival [13]. Notably, Faries et al. [6] demonstrated that patients with late recurrences (lost dormancy) paradoxically exhibit better post‐recurrence survival than those with rapid, early relapses. This may suggest either that long‐dormant melanoma cells inherently lack the aggressive transcriptomic features of early metastases or that the host's breached immune system continues to partially suppress tumor progression post‐relapse. Additionally, weakened host immunity can trigger these dormant cells to reactivate, proliferate, and spread to other organs.

The mechanisms behind tumor dormancy and post‐recurrence survival in late melanoma remain largely undefined, but they are fundamentally rooted in the prolonged, delicate balance between residual cancer cells and host immunity. Available evidence suggests that immune surveillance is central to maintaining disseminated melanoma cells in a dormant or subclinical state; when host immune control weakens, these cells may escape equilibrium, resume proliferation, and eventually metastasize. Thus, it is not immune activation itself that is thought to promote late recurrence, but rather the loss of effective antitumor immune restraint. This concept is supported by prior work linking melanoma dormancy and late relapse to host‐tumor immune equilibrium and its disruption [6, 12, 14, 15, 16]. Despite these biological insights, translating immune equilibrium into specific clinical practice remains elusive.

The translational gap between recognizing the biological threat of immune escape and identifying it in patients directly contributes to the current uncertainty in long‐term disease management. Crowley et al. [7] highlight the imperative for lifelong melanoma surveillance: although overall late recurrence is only 2.4%, approximately 25% of patients who remain disease‐free for 10 years eventually relapse. Because a 10‐year disease‐free interval does not guarantee a cure, this raises critical questions about optimal surveillance durations and the need for clinicopathologic or molecular biomarkers to predict which long‐term survivors remain at risk for immune escape and delayed relapse.

Here, we address a gap in the current literature by exploring the specific patterns of delayed melanoma recurrences. By examining late relapse across diverse patient demographics and primary tumor markers, our findings may offer insights for refining long‐term follow‐up protocols. Importantly, our observations suggest that establishing a complete prognostic picture benefits from a detailed evaluation of the recurrence itself. Because post‐recurrence outcomes can be influenced by the site of relapse, distinguishing between locoregional disease, such as cutaneous, subcutaneous, in‐transit, or nodal events, and distant metastatic spread remains clinically relevant. By highlighting these nuanced anatomic patterns and risk factors, we hope this study contributes to the ongoing discourse on prolonged melanoma surveillance, providing clinicians with additional context to help guide long‐term patient management.

## 2 Methods

### 2.1 Data

We conducted a retrospective cohort study of 30 patients with Stage III or IV melanoma in whom metastatic recurrence was first documented at least 5 years after diagnosis of the primary tumor. Patients were identified from an institutional cohort of approximately 300 individuals treated in the Department of Dermatology and Allergology at the University of Szeged, Hungary. All included patients had available clinicopathologic records and BRAF mutation assessment. Because the clinically relevant temporal variable in this study was recurrence latency, we focused on time to recurrence, captured as progression‐free survival (PFS) in months, rather than reporting exact calendar dates of metastatic events.

Immunotherapy treatment after recurrence was also reviewed descriptively from the clinical records, including immune checkpoint inhibitor use. Specifically, post‐recurrence treatment histories were screened for nivolumab, pembrolizumab, and ipilimumab plus nivolumab, as well as surgery, radiotherapy, chemotherapy, interferon, and targeted therapy. Because of the small sample size and substantial heterogeneity in treatment indication, timing, and sequence, these therapies were not included as formal covariates in the survival models.

### 2.2 Clinicopathologic Variables

The evaluated covariates included age at primary melanoma diagnosis, sex, and BRAF mutation status [17, 18], together with melanoma‐specific variables extracted from histopathology reports and clinical records: primary tumor site, metastatic site localization, melanoma subtype, tumor thickness category, ulceration status, Clark level, Breslow depth, and histologic regression. Initial lymph node excision and initial nodal positivity at the time of primary melanoma treatment were also recorded for descriptive cohort characterization; however, because these data were incomplete for a substantial proportion of patients, they were summarized descriptively and were not entered into the survival models.

Primary tumor site was labeled as trunk, lower limbs, upper limbs, head and neck, acral, mucosal, or uveal. Melanoma subtype was labeled as superficial spreading, nodular, acral lentiginous, or mixed subtype (superficial spreading and nodular). Tumor thickness was categorized as ≤ 1 mm, > 1–2 mm, > 2–4 mm, or > 4 mm. Ulceration and regression were recorded as present or absent, Clark level was recorded on a 1–5 scale, and Breslow depth was analyzed as a continuous variable in millimeters. For clinical interpretation, recurrence pattern was further classified as locoregional or distant; locoregional recurrence comprised local cutaneous/subcutaneous/in‐transit relapse and nodal recurrence, whereas distant recurrence referred to visceral, osseous, central nervous system, or other non‐regional metastatic spread. Because this was a retrospective cohort assembled from routine archival records, immunohistochemical findings, mitotic rate, and neurotropism were not available in a sufficiently complete and standardized manner across the full cohort to support robust statistical analysis and were therefore not included as analytic covariates.

### 2.3 Outcomes

Study outcomes were overall survival (OS), defined as the interval from primary melanoma diagnosis to last follow‐up or death; progression‐free survival (PFS), defined as the interval from primary diagnosis and initial treatment to first documented metastatic recurrence or death; post‐recurrence survival (PRS), defined as the interval from recurrence to death; and vital status at last follow‐up. Histologic regression was defined as partial disappearance of melanoma cells accompanied by host‐response features such as dermal fibrosis, inflammatory infiltrates, melanophages, dilated vessels, epidermal thinning, and/or apoptosis of keratinocytes or melanocytes.

### 2.4 Statistical Analysis

Clinicopathologic covariates included age at primary melanoma diagnosis, sex, BRAF mutation status, primary tumor site, melanoma subtype, tumor thickness, ulceration, Clark level, Breslow thickness, histologic regression, recurrence pattern, and metastatic site burden, as summarized in Table 1. Study endpoints were overall survival (OS), progression‐free survival (PFS), and post‐recurrence survival (PRS). Pairwise relationships between clinicopathologic variables were explored using correlation analysis. Survival distributions were estimated using the Kaplan–Meier method and compared using the log‐rank test; for visualization, age was divided into two groups based on the cohort median of 50 years. Prognostic factors for OS, PFS, and PRS were evaluated using univariable Cox proportional hazards regression, followed by multivariable Cox models selected using the Akaike information criterion. To account for multiple testing, *p*‐values from the Cox models were assessed using the Benjamini‐Krieger‐Yekutieli False Discovery Rate (FDR) procedure, which produced a statistical significance threshold of *p* ≤ 0.047.

**TABLE 1:** Description of the variables monitoring melanoma characteristics in patients.

Variable	Definition	Values
Loc_p	Localization of the diagnosed primary melanoma	0 = trunk 1 = lower limbs 2 = upper limbs 3 = head and neck region 4 = acral region 5 = mucosal surface 6 = uvea
Loc_m	Localization of the diagnosed metastasis	0 = lymph node, 1 = cutaneous 2 = lungs, 3 = liver 4 = central nervous system 5 = bone, 6 = peritoneum 7 = ovary, 8 = parotid 9 = small intestine 10 = large intestine 11 = salivary glands 12 = spleen 13 = gallbladder, 14 = kidney 15 = adrenal, 16 = satellite 17 = subcutaneous 18 = muscle, 19 = thyroid 20 = cartilage, 21 = heart 22 = thorax, 23 = gastric 24 = pelvis, 25 = intestines 26 = breast, 27 = pleura 28 = scrotum, 29 = pancreas 30 = mesentery, 31 = sinus 32 = choroid, uvea 33 = esophagus
Loc_m_cnt	The total number of unique metastasis locations for each patient	A count: values observed in the data range from 1 to 7
Type	Subtype of malignant melanoma	Takes values 1, 2 or 3 depending on the subtype of malignant melanoma: 1 = superficial spreading melanoma (SSM), 2 = nodular melanoma (NM), 3 = acral lentiginous melanoma (ALM)
*T*	Thickness of melanoma	1 = ≤ 1 mm 2 = > 1.0 to 2.0 mm 3 = > 2.0 to 4.0 mm 4 = > 4 mm
*U*	Ulceration	Indicator variable: 1 in case of ulceration 0 otherwise
Clark	The depth stage of melanoma as it penetrates the skin	1 = Limited to the epidermis, the skin's surface layer 2 = Entered the papillary dermis, the outermost section of the dermis beneath the epidermis 3 = Spread throughout the papillary dermis and reached the next, deeper dermal layer 4 = Extended into the reticular dermis, the next deeper layer 5 = Penetrated the subdermal fat tissue beneath the dermis
Breslow's depth	The depth of melanoma is measured starting from the topmost portion of the granular layer in the epidermis, or from the base of any existing surface ulcers, extending to the deepest invasive cell at the expansive basal section of the tumor, located in the dermal or subcutaneous layer.	Breslow depth is a continuous variable that measures depth in mm
Regression	Histologically, it is characterized by a diverse reduction in melanoma cells, alongside a host response that includes dermal fibrosis, inflammatory infiltrate, presence of melanophages, dilated blood vessels, thinning of the epidermis, and/or apoptosis of keratinocytes or melanocytes	0 = No 1 = Yes

## 3 Results

The study cohort comprised 30 patients with delayed melanoma recurrence (Table 2), including 18 women and 12 men, with a mean age of 50.2 years (range, 25–76 years). The mean overall survival (OS) was 168.7 months (range, 60–378), the mean progression‐free survival (PFS) was 130.4 months (range, 60–333), and the mean post‐recurrence survival (PRS) was 43.7 months (range, 2–161). Mean Breslow thickness, available in 28 patients, was 2.61 mm (range, 0.608–10.032). Most primary melanomas were Clark Level III or IV (80.0%, *n* = 24), 53.3% (*n* = 16) were ulcerated, and the trunk was the most common primary site (50.0%, *n* = 15), followed by the upper limbs (23.3%, *n* = 7), lower limbs (16.7%, *n* = 5), and the head and neck region (6.7%, *n* = 2); one case had missing primary‐site data. BRAF mutations were present in 50.0% of tumors (*n* = 15), and the most common melanoma subtype was superficial spreading melanoma (50.0%, *n* = 15). Histologic regression was present in 5 patients (16.7%), absent in 20 (66.7%), and unavailable in 5 (16.7%). Initial lymph node excision was documented in 8 patients (26.7%), whereas initial nodal positivity was documented in 5 (16.7%); nodal positivity data were unavailable in 21 patients. At recurrence, 8 patients (26.7%) developed locoregional recurrence, defined as local cutaneous, subcutaneous, in‐transit, or nodal relapse, whereas 21 patients (70.0%) developed distant metastasis; one case had incomplete localization data. During follow‐up, eight patients died, including two women and six men. Detailed survival data and melanoma characteristics are available in Table 3.

**TABLE 2:** Gender and age characteristics of the 30 patients in the cohort study.

Patient characteristics, *n* = 30	
Males	12
Females	18
Mean age of the patients	The average age is 50.2 years (range spanning from 25 to 76 years)

**TABLE 3:** Survival and melanoma characteristics of the 30 patients included. OS denotes overall survival, PFS represents progression‐free survival, PRS signifies post‐recurrence survival, SSM refers to superficial spreading melanoma, NM indicates nodular melanoma, and ALM stands for acral lentiginous melanoma.

Survival & melanoma characteristics	
Mean OS (months)	168.7, range 60–378
Mean PFS (months)	130.4, range 60–333
Mean PRS (months)	43.7, range 2–161
Live	
Yes	22 (73%)
No	8 (27%)
Mean tumor thickness (mm) (*n* = 28)	2.61, range 0.608–10.032
Clark level	
I–II	4 (13%)
III	18 (60%)
IV	6 (20%)
V	1 (3%)
N/A	1 (3%)
Ulceration	
Yes	16 (53%)
No	10 (33%)
N/A	4 (13%)
Primary site	
Head/neck	2 (7%)
Upper limbs	7 (23%)
Lower limbs	5 (17%)
Trunk	15 (50%)
N/A	1 (3%)
BRAF	
Yes	15 (50%)
No	13 (43%)
N/A	2 (7%)
Melanoma type	
SSM	15 (50%)
NM	9 (30%)
ALM	1 (3%)
SSM & NM	2 (7%)
N/A	3 (10%)
Regression	
Yes	5 (17%)
No	20 (67%)
N/A	5 (17%)
Localization	
Local	8 (27%)
Distant	21 (70%)
N/A	1 (3%)
Initial lymph node excision	
Yes	8 (27%)
No	21 (70%)
N/A	1 (3%)
Initial lymph node positive	
Yes	5 (17%)
No	4 (13%)
N/A	21 (70%)
Mean Loc_m_cnt (total number of unique metastasis locations)	2.367, range 1–7

To characterize the clinical course following these delayed relapses, we reviewed the post‐recurrence therapies administered and identified immune checkpoint inhibitor exposure in 10 patients. Seven patients received nivolumab‐containing regimens, including 1 regimen combined with ipilimumab, and three patients received pembrolizumab. Immunotherapy was used in heterogeneous settings, including adjuvant treatment after resection of late recurrence and treatment of disseminated metastatic disease, often after prior surgery, radiotherapy, chemotherapy, or BRAF/MEK‐targeted therapy. Treatment was discontinued because of immune‐related toxicity in 3 patients, specifically hypophysitis, pneumonitis, and pancreatitis. Evidence of disease control was documented in 4 patients, including 1 with a good radiologic response, 1 with total remission, 1 who remained free of confirmed recurrence during recorded follow‐up after adjuvant immunotherapy, and 1 with no metabolically active residual lesion on subsequent PET/CT. In contrast, 2 patients developed progression after immunotherapy, while 4 had insufficient follow‐up to assess response.

Having established the clinical and treatment profiles of the cohort, we shifted our focus to identifying statistical relationships among the variables using a correlation matrix (Table 4). As anticipated, a strong positive correlation was observed between T category and Breslow thickness (*r* = 0.78). Although both metrics reflect tumor depth, a perfect correlation is precluded because the T category represents a four‐level ordinal grouping of the continuous Breslow measurement. This transformation compresses the continuous data into broad categories, which inherently attenuates the Pearson correlation coefficient. Furthermore, age, sex, and Clark level were significantly correlated with the primary survival outcomes, progression‐free survival (PFS) and overall survival (OS).

**TABLE 4:** Pearson correlation matrix (color is deeper green as we move to + 1.00, and color is deeper red as we move towards − 1.00).

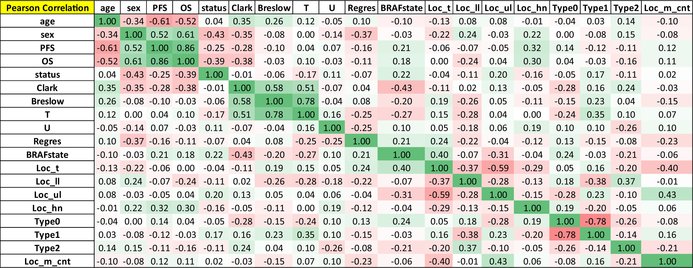

To evaluate the prognostic significance of individual clinicopathologic variables, we performed univariable Cox proportional hazards analyses for OS, PFS, and PRS (Table 5). Most clinicopathologic variables were not significantly associated with survival outcomes in this cohort. For OS, female sex was the only significant covariate (HR 0.055, 95% CI 0.01–0.47; *p* = 0.008), whereas age, primary site, metastatic site burden, melanoma subtype, T category, ulceration, Clark level, Breslow thickness, histologic regression, BRAF status, recurrence pattern, and PRS were not significant (Table 5a). For PFS, age (HR 1.067, 95% CI 1.00–1.10; *p* = 0.001), female sex (HR 0.223, 95% CI 0.10–0.56; *p* = 0.001), and Clark level (HR 2.075, 95% CI 1.00–4.10; *p* = 0.036) were significant in univariable analysis, whereas primary site, metastatic site burden, melanoma subtype, T category, ulceration, Breslow thickness, histologic regression, BRAF status, recurrence pattern, and PRS were not (Table 5b). For PRS, no covariate met the prespecified significance threshold, although sex showed a near‐significant association (HR 0.202, 95% CI 0.04–1.00; *p* = 0.056) (Table 5c).

**TABLE 5:** Univariate analysis outcomes related to melanoma survival data which encompasses (a) overall survival, (b) progression‐free survival, and (c) survival following recurrence. *p*‐values that fall below the significance threshold of 0.047, after adjustment for multiple testing, are highlighted in bold.

Variable	Beta	HR (95% CI for HR)	*p*
(a) Overall survival			
Age	0.051	1.052 (0.99–1.10)	0.120
Sex	−2.900	0.055 (0.01–0.47)	**0.008**
Loc_p	−0.074	0.929 (0.47–1.80)	0.830
Loc_m_cnt	−0.050	0.951 (0.69–1.30)	0.760
Type	0.100	1.105 (0.28–4.40)	0.890
*T*	−0.500	0.607 (0.27–1.40)	0.230
*U*	0.390	1.477 (0.30–7.30)	0.640
Clark	0.290	1.336 (0.45–4.00)	0.600
Breslow	−0.032	0.969 (0.75–1.20)	0.800
Regres	0.100	1.105 (0.13–9.20)	0.920
BRAFstate	0.720	2.054 (0.41–10.00)	0.380
loc_rec	−0.930	0.395 (0.048–3.20)	0.390
PRS	−0.014	0.986 (0.96–1.00)	0.270
(b) Progression‐free survival			
Age	0.065	1.067 (1.00–1.10)	**0.001**
Sex	−1.500	0.223 (0.10–0.56)	**0.001**
Loc_p	−0.190	0.827 (0.58–1.20)	0.290
Loc_m_cnt	−0.068	0.934 (0.79–1.10)	0.420
Type	0.440	1.553 (0.77–3.20)	0.220
*T*	−0.160	0.852 (0.54–1.40)	0.500
*U*	−0.200	0.819 (0.37–1.80)	0.610
Clark	0.730	2.075 (1.00–4.10)	**0.036**
Breslow	0.011	1.011 (1.00–1.10)	0.084
Regres	0.680	1.974 (0.73–5.30)	0.180
BRAFstate	−0.510	0.600 (0.28–1.30)	0.190
loc_rec	0.110	1.116 (0.50–2.50)	0.790
PRS	0.002	1.002 (0.99–1.00)	0.710
(c) Post‐recurrence survival			
Age	0.015	1.015 (0.96–1.10)	0.600
Sex	−1.600	0.202 (0.04–1.00)	0.056
Loc_p	0.018	1.018 (0.51–2.00)	0.960
Loc_m_cnt	0.009	1.009 (0.74–1.40)	0.950
Type	−0.200	0.819 (0.20–3.40)	0.790
*T*	−0.330	0.719 (0.31–1.70)	0.450
*U*	0.920	2.509 (0.49–13.00)	0.270
Clark	0.100	1.105 (0.43–2.80)	0.830
Breslow	−0.024	0.976 (0.89–1.10)	0.630
Regres	−0.560	0.571 (0.07–4.70)	0.600
BRAFstate	0.930	2.535 (0.51–13.00)	0.250
loc_rec	−1.000	0.368 (0.04–2.90)	0.330

To further evaluate and visualize the key findings from our univariable models, we assessed survival distributions across specific covariate levels using the Kaplan–Meier method and log‐rank test. We found a significant (*p* < 0.001) difference in overall survival (OS) between sexes, with men having a mean OS of 8 years and women 15 years (Figure 1a). For progression‐free survival (PFS), females showed a significant survival benefit (*p* < 0.001), with an average PFS of 11 years compared to 6 years for males (Figure 1b). Age was also identified as a prognostic factor for PFS; individuals under 50 years experienced a significantly longer time to recurrence (*p* < 0.001) than those over 50. The 50‐year cutoff was chosen because it reflects the median age in our clinical data (Figure 1c).

**FIGURE 1:**
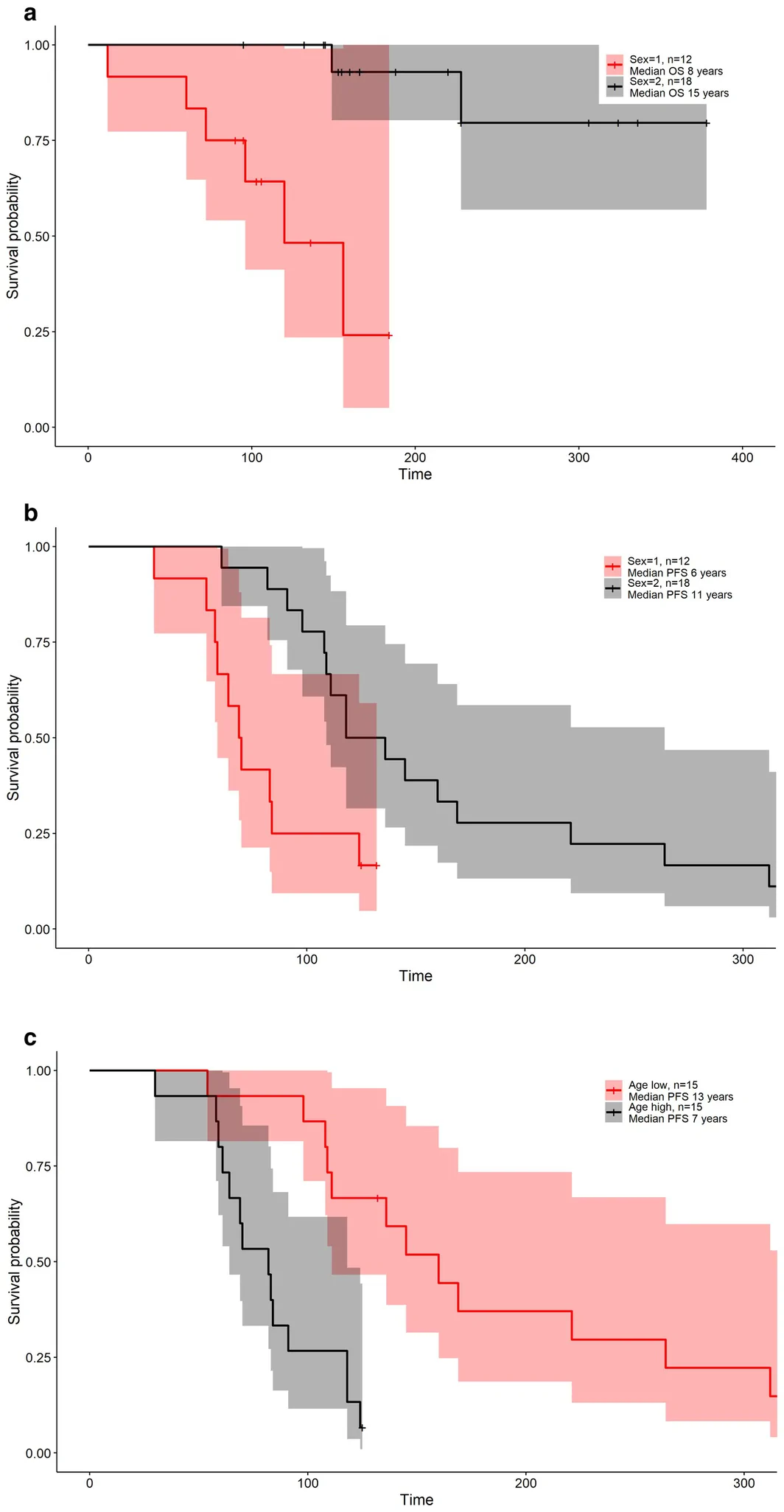
Kaplan–Meier survival analyses identified significant associations between clinical variables and melanoma survival outcomes. (a) Overall survival by sex, comparing males (red line, red shaded area) and females (black line, gray shaded area) (*p* < 0.001). (b) Progression‐free survival by sex, comparing males (red line, red shaded area) and females (black line, gray shaded area) (*p* < 0.001). (c) Progression‐free survival by age group, comparing low age (red line, red shaded area) and high age (black line, gray shaded area) (*p* < 0.001).

Finally, given that several covariates were significantly correlated with the outcome variables, we constructed multivariable models to isolate their independent prognostic value. We selected the optimal model according to the Akaike information criterion (AIC). In the multivariable analysis for PFS (Table 6), increasing age remained associated with shorter PFS (HR 1.072, 95% CI 1.03–1.12; *p* = 0.001), whereas female sex was associated with longer PFS (HR 0.364, 95% CI 0.14–0.93; *p* = 0.035). Histologic regression (HR 3.557, 95% CI 1.04–12.17; *p* = 0.043) and BRAF status (HR 0.413, 95% CI 0.18–0.94; *p* = 0.034) were also retained in the final AIC‐selected model. However, these latter associations should be interpreted with caution because regression was observed in only a small subset of patients, and neither regression nor BRAF status was significant in univariable analysis. In both OS and PRS, multivariable analyses identified at most one significant predictor.

**TABLE 6:** Results from the multivariate analysis for progression‐free survival are presented. *p*‐values that fall below the significance threshold, adjusted for multiple testing to 0.047, are highlighted in bold.

Progression‐free survival			
Variables	Beta	HR (95% CI for HR)	*p*
Age	0.069	1.072 (1.03–1.12)	**0.001**
Sex	−1.011	0.364 (0.14–0.93)	**0.035**
Regres	1.269	3.557 (1.04–12.17)	**0.043**
BRAFstate	−0.884	0.413 (0.18–0.94)	**0.034**

## 4 Discussion

Our study investigates clinicopathologic factors associated with survival after delayed melanoma recurrence, a critical yet understudied area of clinical importance. Existing guidelines generally do not advocate for routine follow‐ups beyond 5 years post‐initial diagnosis and complete resection, aside from continuing with yearly dermatological evaluations [1]. This is concerning, as we identified specific predictive factors that increase the risk for late recurrence, revealing a crucial gap in current recommendations. Notably, melanoma survivors already face a significant risk of developing another melanoma or different types of skin cancer [12].

Several well‐established prognostic factors, including tumor thickness, ulceration, invasion level, age, primary tumor site, and recurrence pattern, are recognized in melanoma management [19, 20]. In our delayed‐recurrence cohort, these features provide important pathologic context for the observed outcomes. Mean Breslow thickness was 2.61 mm; ulceration was present in more than half of tumors; the trunk was the most common primary site; and most late recurrences were distant rather than locoregional. Although these variables did not all emerge as independent predictors in our models, they remain clinically relevant determinants of the cohort and help contextualize the biology of late relapse. By contrast, immunohistochemical findings, mitotic rate, and neurotropism were not available in a sufficiently standardized manner across the archival records to permit robust statistical analysis and should therefore be addressed in future larger studies with more comprehensive pathology annotations.

Beyond the intrinsic histopathologic characteristics of the primary tumor, it is important to recognize that contemporary management of these patients increasingly involves complex systemic treatments. Immune checkpoint inhibitor therapy constituted an important component of treatment in this cohort. These agents were used in diverse clinical contexts, including the adjuvant setting after resection and as systemic treatment for advanced recurrent disease, frequently alongside or after surgery, radiotherapy, chemotherapy, and targeted therapy. In addition, some immunotherapy courses were discontinued because of disease progression or immune‐related toxicities, further underscoring the heterogeneity of post‐recurrence management in this cohort. Given the marked heterogeneity in treatment indication, timing, and sequencing, the cohort was not suitable for formal inference regarding the effect of immunotherapy on outcomes. Nonetheless, the presence of checkpoint inhibitor treatment within the cohort underscores that contemporary management of late melanoma recurrence increasingly occurs in the era of modern systemic therapy and should be considered when interpreting survival findings.

Although current systemic therapies target active disease, the long latency period before late recurrences highlights the importance of exploring the fundamental mechanisms of tumor dormancy. In melanoma, dormant disease may reflect either cellular quiescence of disseminated tumor cells or a state of dynamic equilibrium in which limited proliferation is counterbalanced by apoptosis and immune‐mediated control. This equilibrium may persist for years and can be disrupted by immunosenescence, microenvironmental changes, angiogenic switching, or loss of effective immune surveillance, thereby permitting clinically overt metastatic growth [13, 21]. The observation by Faries et al. [6] that survival curves for early and late recurrence initially overlap and subsequently diverge is consistent with the concept that late relapse may reflect reactivation of previously restrained disease rather than merely delayed detection. Our findings that younger age and female sex were associated with longer PFS may suggest, though not prove, more durable host control of dormant melanoma cells. Taken together, these considerations support the biological plausibility of late relapse and reinforce the rationale for prolonged surveillance, particularly in patient groups at risk for delayed recurrence. This would permit early intervention for late recurrence, thus potentially improving long‐term outcomes.

Given the biological plausibility of prolonged dormancy, identifying which patient demographics are most susceptible to eventual tumor reactivation is crucial. Among these predictive variables, age stands out as a significant determinant of the time between initial diagnosis and recurrence. Younger patients exhibit significantly longer PFS than older patients, consistent with earlier research [3, 4, 6, 7, 8, 22, 23, 24, 25]. This can be partially attributed to declining immune surveillance with age, which is less effective at keeping tumors dormant in older patients [26]. Regarding post‐recurrence survival, Reintgen et al. [20] identified younger age as a predictor of survival. We were unable to demonstrate this post‐recurrence survival advantage.

Sex is another key variable. Females in our cohort experienced later recurrences of melanoma, a finding corroborated by prior literature [2, 6, 15, 19]. Several theories have been proposed to explain this trend. Female hormones might be involved, as well as immunocompetence, which decreases with age and declines earlier in males than in females [11, 27]. Additionally, females exhibit less attenuation of adaptive immune responses and are less prone to skin cancer, likely because they experience less ultraviolet light‐induced immunosuppression [28].

Although demographic factors such as age and sex significantly influenced PFS, our analysis revealed that most conventional clinicopathologic variables did not independently predict survival in this delayed‐recurrence cohort. Primary site, melanoma subtype, T category, ulceration, Breslow thickness, recurrence pattern, and metastatic site burden were not significant in the univariable models, and no covariate met the significance threshold for PRS. These largely negative findings are still informative because they underscore the difficulty of identifying robust and reproducible prognostic markers in small cohorts with delayed recurrence and are consistent with prior literature reporting inconsistent clinicopathologic predictors of late relapse [4, 6, 10, 20, 22, 24, 29].

Despite these largely negative findings for conventional markers, specific histopathologic and molecular features, namely histologic regression and BRAF mutation status, warranted closer examination. In our multivariable model, histologic regression was associated with shorter PFS within this delayed‐recurrence cohort. Regression reflects the partial replacement of melanoma with fibrosis, inflammation, melanophages, and vascular changes, but its prognostic significance remains controversial in the literature [30, 31]. Our findings regarding regression warrant particularly cautious interpretation. In our cohort, regression was present in only 5 of 30 patients and was unavailable in 5 others; it was not significant in univariable analyses of OS, PFS, or PRS. Although regression entered the AIC‐selected multivariable PFS model, this association should be regarded as hypothesis‐generating rather than definitive. However, these results support retaining regression as a clinically relevant histopathologic variable in future, larger studies focused specifically on late recurrence.

Similarly, the association between BRAF status and PFS should be interpreted as exploratory and hypothesis‐generating. Although BRAF status was retained in the multivariable model, it was not significant in univariable analysis and therefore requires validation in larger cohorts. To date, no association has been identified between a BRAF mutation and the development of late metastasis. Only speculation can be made regarding this connection. Analysis of BRAF mutations and their associations with clinicopathological features of primary malignant melanoma showed that BRAF mutations were associated with younger age and localization to the trunk [32, 33]. These features are consistent with the characteristics of patients with late metastasis reported in the literature. BRAF‐mutant melanoma patients already have the advantage of more effective treatments due to the availability of drugs that specifically target BRAF‐mutant cells, which may influence the onset of metastases.

Beyond PFS, understanding the factors that dictate patient outcomes after a late recurrence has manifested is equally critical for clinical management. Regarding overall and post‐recurrence survival, factors such as tumor thickness, invasion level, age, primary tumor site, and recurrence pattern are well‐established [19, 20, 34, 35, 36]. Our data indicates that female sex may be associated with longer PRS, consistent with its association with longer PFS and OS, although this finding barely missed statistical significance (*p* = 0.056). Crowley et al. [7] identified a survival difference based on the primary tumor site through univariate analysis and noted that PRS depended solely on the metastasis location. The finding that late regional recurrences have better outcomes than late distant recurrences is supported by the literature [5, 20, 24, 25, 37]. Tsao et al. [5] estimated that the probability of survival (5 years after recurrence) was 0.8 for regional disease and 0.2 for distant disease. Faries et al. [6] found that patients with late recurrence had better survival after recurrence than those with early recurrence. They assessed various independent prognostic factors for post‐recurrence survival, including Breslow thickness, primary site, lymph node status, recurrence stage, and whether the recurrence was late or early. Additionally, previous studies suggest that the interval between diagnosis and first recurrence is not significantly associated with survival outcomes [8, 22, 24, 25].

A notable contribution of this study is the multivariate approach. As the literature indicates, we also observed several covariates to be related to the outcome variables in the correlation analysis and the univariate survival analysis. Moreover, the covariates can be correlated with one another. Therefore, a multivariate approach can simultaneously provide insights into the associations between multiple covariates and the outcome while controlling for their interrelationships. Taken together, the results of this study add to the growing literature on risk factors for late melanoma recurrence. These findings may help identify subgroups that could benefit from closer long‐term surveillance, including older patients and male patients, and thereby inform future follow‐up recommendations.

Although this multivariate analysis yielded valuable insights into at‐risk subgroups that may benefit from extended surveillance, these findings must be interpreted in the light of several study limitations. First and foremost, the small sample size and the resulting low number of events could introduce bias and reduce the statistical power of our findings, potentially leading to the omission of relevant predictors. The small sample size, however, is due to the rarity of finding patients with the desired inclusion criteria. Furthermore, the limited follow‐up duration might underestimate the true extent of late recurrence rates, as the full spectrum of this phenomenon may not have been captured during the study period. Referral bias is also a concern, as patients in the dataset may not be fully representative of the broader population, which could affect the generalizability of our conclusions. In the absence of a comparable control group, drawing direct comparisons with populations that do not experience late recurrences remains challenging. An additional limitation is that certain histopathologic variables of potential prognostic interest, including immunohistochemical findings, mitotic rate, and neurotropism, were not uniformly available in the retrospective records and therefore could not be analyzed in a standardized manner. Finally, treatment heterogeneity represents an additional limitation. Review of the clinical records identified multiple patients treated with immune checkpoint inhibitors, including nivolumab‐, pembrolizumab‐, and combined ipilimumab plus nivolumab‐based regimens, administered in both adjuvant and metastatic settings. Because the number of treated patients was limited and the timing, sequencing, and indications for immunotherapy varied substantially, treatment‐specific outcome analyses were not performed.

In summary, this study underscores the imperative to re‐evaluate the notion of “cure” in cutaneous melanoma cases. Although conventional guidelines often consider a 10‐year disease‐free period indicative of cure, our findings suggest that a subset of patients remains at risk of late recurrence beyond this threshold. Notably, our analysis identified associations with OS and PFS, with more favorable outcomes observed among women and younger patients, whereas no clinicopathologic factor met the prespecified significance threshold for PRS. This highlights vulnerable groups, such as elderly males, that may warrant heightened vigilance for late recurrence. The study underscores the necessity for prolonged patient follow‐up, even beyond the conventional 10‐year mark, to ensure timely intervention and appropriate treatment. As the treatment landscape continues to evolve and the complexities of late melanoma recurrence are better understood, healthcare providers are urged to remain vigilant and extend monitoring periods. Although this work lays a foundation, larger‐scale studies are crucial for refining risk stratification, validating extended monitoring approaches, and clarifying which patient subgroups may benefit from tailored long‐term surveillance. The broader goal is to refine melanoma management and optimize patient outcomes in this under‐explored area of clinical concern.

## Author Contributions

**Kaspar Itin:** conceptualization, validation, formal analysis, writing – original draft. **Grzegorz Sarek:** writing – original draft, project administration. **Elisabeth Roider:** conceptualization, validation, formal analysis, funding acquisition, writing – original draft, supervision, project administration. **Lara Valeska Maul:** writing – review and editing. **Alina. M. Torrez:** validation, formal analysis, writing – review and editing. **Alexander A. Navarini:** funding acquisition. **Babak Saravi:** writing – review and editing. **Rebecca Lapides:** conceptualization, validation, formal analysis, writing – review and editing. **Leticia Szadai:** resources. **Michael Wang‐Evers:** writing – review and editing. **Beata Kun:** resources, methodology, validation, formal analysis, writing – original draft. **Eszter Baltas:** writing – review and editing. **Soumya Paria:** methodology, validation, formal analysis, writing – original draft. **Judit Olàh:** writing – review and editing. **Dieter Manstein:** writing – review and editing. **Istvan B. Németh:** conceptualization, resources, methodology, validation, formal analysis, writing – original draft, supervision. **Rolland Gyulai:** writing – review and editing. **Peter Hollo:** writing – review and editing. **Kaustubh Adhikari:** conceptualization, methodology, validation, formal analysis, writing – original draft, supervision.

## Funding

Work performed in the Roider laboratory was supported by the Swiss National Science Foundation (SNSF; Grant No. 310030_220213/1).

## Ethics Statement

The study was conducted in accordance with the guidelines of the Declaration of Helsinki. The ethics approval number is MEL‐PROTEO‐001 (University of Szeged, Hungary). This research protocol has been approved by the Ministry of Human Resources (Hungary) (EMMI), Deputy State Secretariat for National Chief Medical Officer Tasks, Department of Health Administration (NNK/NNGYK), with approval number 4463‐6/2018/EÜIG. The regional ethics approval number, granted by the Institutional Research Ethics Committee (IKEB), is 4248/20‐77/2018‐SZTE. The subject leader is also a participant in the MEL‐RETRO‐001 license.

## Consent

Written informed consent was obtained by the study participants.

## Conflicts of Interest

The authors declare no conflicts of interest.

## Data Availability

The data used to generate the findings of this study were obtained from the MedSolution system of the Department of Dermatology and Allergology at the University and Clinics of Szeged, Hungary, and are not publicly available to protect patient privacy.

## References

[1] C. Garbe , T. Amaral , K. Peris , et al., “European Consensus‐Based Interdisciplinary Guideline for Melanoma. Part 1: Diagnostics: Update 2022,” European Journal of Cancer 170 (2022): 236–255. 10.1016/j.ejca.2022.03.008 35570085

[2] H. A. Briele , C. W. Beattie , S. G. Ronan , P. K. Chaudhuri , and T. K. Das Gupta , “Late Recurrence of Cutaneous Melanoma,” Archives of Surgery 118, no. 7 (1983): 800–803. 10.1001/archsurg.1983.01390070012003 6860127

[3] G. Hansel , J. Schönlebe , G. Haroske , and U. Wollina , “Late Recurrence (10 Years or More) of Malignant Melanoma in South‐East Germany (Saxony). A Single‐Centre Analysis of 1881 Patients With a Follow‐Up of 10 Years or More,” Journal of the European Academy of Dermatology and Venereology 24, no. 7 (2010): 833–836. 10.1111/j.1468-3083.2009.03536.x 20070457

[4] J. A. Brauer , C. C. Wriston , A. B. Troxel , et al., “Characteristics Associated With Early and Late Melanoma Metastases,” Cancer 116, no. 2 (2010): 415–423. 10.1002/cncr.24724 19918926

[5] H. Tsao , A. B. Cosimi , and A. J. Sober , “Ultra‐Late Recurrence (15 Years or Longer) of Cutaneous Melanoma,” Cancer 79, no. 12 (1997): 2361–2370. 9191524

[6] M. B. Faries , S. Steen , X. Ye , M. Sim , and D. L. Morton , “Late Recurrence in Melanoma: Clinical Implications of Lost Dormancy,” Journal of the American College of Surgeons 217, no. 1 (2013): 27–34. 10.1016/j.jamcollsurg.2013.03.007 PMC3731060 23643694

[7] N. J. Crowley and H. F. Seigler , “Late Recurrence of Malignant Melanoma. Analysis of 168 Patients,” Annals of Surgery 212, no. 2 (1990): 173–177. 10.1097/00000658-199008000-00010 PMC1358053 2375648

[8] M. H. Schmid‐Wendtner , J. Baumert , M. Schmidt , et al., “Late Metastases of Cutaneous Melanoma: An Analysis of 31 Patients,” Journal of the American Academy of Dermatology 43, no. 4 (2000): 605–609. 10.1067/mjd.2000.107234 11004614

[9] P. Shen , J. M. Guenther , L. A. Wanek , and D. L. Morton , “Can Elective Lymph Node Dissection Decrease the Frequency and Mortality Rate of Late Melanoma Recurrences?,” Annals of Surgical Oncology 7, no. 2 (2000): 114–119. 10.1007/s10434-000-0114-x 10761789

[10] M. P. Callaway and J. C. Briggs , “The Incidence of Late Recurrence (Greater Than 10 Years); an Analysis of 536 Consecutive Cases of Cutaneous Melanoma,” British Journal of Plastic Surgery 42, no. 1 (1989): 46–49. 10.1016/s0007-1226(89)90111-2 2917216

[11] J. Yan , J. M. Greer , R. Hull , et al., “The Effect of Ageing on Human Lymphocyte Subsets: Comparison of Males and Females,” Immunity & Ageing 7 (2010): 4. 10.1186/1742-4933-7-4 PMC2858100 20233447

[12] D. P. Tahery and R. L. Moy , “Lack of Predictive Factors in Late Recurrence of Stage I Melanoma,” International Journal of Dermatology 31, no. 9 (1992): 629–631. 10.1111/j.1365-4362.1992.tb03980.x 1459759

[13] L. Ossowski and J. A. Aguirre‐Ghiso , “Dormancy of Metastatic Melanoma,” Pigment Cell & Melanoma Research 23, no. 1 (2010): 41–56. 10.1111/j.1755-148X.2009.00647.x PMC2821074 19843243

[14] J. R. Yarnold and J. P. Cochrane , “Malignant Melanoma: Significance of Disease‐Free Interval,” British Journal of Radiology 53, no. 635 (1980): 1068–1070. 10.1259/0007-1285-53-635-1068 7426933

[15] D. Raderman , S. Giler , A. Rothem , and M. Ben‐Bassat , “Late Metastases (Beyond Ten Years) of Cutaneous Malignant Melanoma. Literature Review and Case Report,” Journal of the American Academy of Dermatology 15, no. 2 Pt 2 (1986): 374–378. 10.1016/s0190-9622(86)70182-5 3734186

[16] N. W. Pearlman , T. J. Takach , W. A. Robinson , J. Ferguson , and A. L. Cohen , “A Case‐Control Study of Late Recurrence of Malignant Melanoma,” American Journal of Surgery 164, no. 5 (1992): 458–460. 10.1016/s0002-9610(05)81180-8 1443369

[17] A. Alqathama , “BRAF in Malignant Melanoma Progression and Metastasis: Potentials and Challenges,” American Journal of Cancer Research 10, no. 4 (2020): 1103–1114. PMC7191094 32368388

[18] J. D. Wolchok , V. Chiarion‐Sileni , R. Gonzalez , et al., “Long‐Term Outcomes With Nivolumab Plus Ipilimumab or Nivolumab Alone Versus Ipilimumab in Patients With Advanced Melanoma,” Journal of Clinical Oncology 40, no. 2 (2022): 127–137. 10.1200/JCO.21.02229 PMC8718224 34818112

[19] C. Balch , “An Analysis of Prognostic Factors in 8500 Patients With Cutaneous Melanoma,” Cutan Melanoma, (1992), 163–187.

[20] D. S. Reintgen , C. Cox , C. L. Slingluff , and H. F. Seigler , “Recurrent Malignant Melanoma: The Identification of Prognostic Factors to Predict Survival,” Annals of Plastic Surgery 28, no. 1 (1992): 45–49. 10.1097/00000637-199201000-00013 1642405

[21] R. Reschke , K. Dumann , and M. Ziemer , “Risk Stratification and Clinical Characteristics of Patients With Late Recurrence of Melanoma (> 10 Years),” Journal of Clinical Medicine 11, no. 7 (2022): 2026. 10.3390/jcm11072026 PMC9000041 35407631

[22] E. Sarac , J. Wilhelmi , I. Thomas , et al., “Late Recurrence of Melanoma After 10 Years ‐ Is the Course of the Disease Different From Early Recurrences?,” Journal of the European Academy of Dermatology and Venereology 34, no. 5 (2020): 977–983. 10.1111/jdv.16106 31758713

[23] E. Levy , M. K. Silverman , K. A. Vossaert , et al., “Late Recurrence of Malignant Melanoma: A Report of Five Cases, a Review of the Literature and a Study of Associated Factors,” Melanoma Research 1, no. 1 (1991): 63–67. 1822771

[24] H. M. Shaw , C. W. Beattie , W. H. McCarthy , and G. W. Milton , “Late Relapse From Cutaneous Stage I Malignant Melanoma,” Archives of Surgery 120, no. 10 (1985): 1155–1159. 10.1001/archsurg.1985.01390340053010 4038058

[25] A. Peters , A. Lippold , and M. Hundeiker , “First Melanoma Metastases After 10 Years and More of Remission,” Der Hautarzt 48, no. 5 (1997): 311–317. 10.1007/s001050050588 9303904

[26] L. Malaguarnera , E. Cristaldi , and M. Malaguarnera , “The Role of Immunity in Elderly Cancer,” Critical Reviews in Oncology/Hematology 74, no. 1 (2010): 40–60. 10.1016/j.critrevonc.2009.06.002 19577481

[27] C. M. Balch , S. J. Soong , J. E. Gershenwald , et al., “Prognostic Factors Analysis of 17,600 Melanoma Patients: Validation of the American Joint Committee on Cancer Melanoma Staging System,” Journal of Clinical Oncology 19, no. 16 (2001): 3622–3634. 10.1200/JCO.2001.19.16.3622 11504744

[28] M. R. Roh , P. Eliades , S. Gupta , J. M. Grant‐Kels , and H. Tsao , “Cutaneous Melanoma in Women,” International Journal of Women's Dermatology 3, no. Suppl 1 (2017): S11–S15. 10.1016/j.ijwd.2017.02.003 PMC5419022 28492033

[29] C. L. Slingluff , R. K. Dodge , W. E. Stanley , and H. F. Seigler , “The Annual Risk of Melanoma Progression. Implications for the Concept of Cure,” Cancer 70, no. 7 (1992): 1917–1927. 10.1002/1097-0142(19921001)70:7<1917::aid-cncr2820700719>3.0.co;2-5 1525767

[30] F. Tas and K. Erturk , “Presence of Histological Regression as a Prognostic Factor in Cutaneous Melanoma Patients,” Melanoma Research 26, no. 5 (2016): 492–496. 10.1097/CMR.0000000000000277 27380112

[31] C. Requena , R. Botella‐Estrada , V. Traves , E. Nagore , S. Almenar , and C. Guillén , “Problems in Defining Melanoma Regression and Prognostic Implication,” Actas Dermo‐Sifiliográficas 100, no. 9 (2009): 759–766. 19889297

[32] M. V. Heppt , T. Siepmann , J. Engel , et al., “Prognostic Significance of BRAF and NRAS Mutations in Melanoma: A German Study From Routine Care,” BMC Cancer 17, no. 1 (2017): 536. 10.1186/s12885-017-3529-5 PMC5553744 28797232

[33] S. Y. Kim , S. N. Kim , H. J. Hahn , Y. W. Lee , Y. B. Choe , and K. J. Ahn , “Metaanalysis of BRAF Mutations and Clinicopathologic Characteristics in Primary Melanoma,” Journal of the American Academy of Dermatology 72, no. 6 (2015): 1036–1046.e2. 10.1016/j.jaad.2015.02.1113 25819940

[34] F. Meier , S. Will , U. Ellwanger , et al., “Metastatic Pathways and Time Courses in the Orderly Progression of Cutaneous Melanoma,” British Journal of Dermatology 147, no. 1 (2002): 62–70. 10.1046/j.1365-2133.2002.04867.x 12100186

[35] U. Leiter , F. Meier , B. Schittek , and C. Garbe , “The Natural Course of Cutaneous Melanoma,” Journal of Surgical Oncology 86, no. 4 (2004): 172–178. 10.1002/jso.20079 15221923

[36] C. Garbe , P. Büttner , J. Bertz , et al., “Primary Cutaneous Melanoma. Prognostic Classification of Anatomic Location,” Cancer 75, no. 10 (1995): 2492–2498. 10.1002/1097-0142(19950515)75:10<2492::aid-cncr2820751015>3.0.co;2-w 7736393

[37] J. A. Leman and R. M. Mac Kie , “Late (> 10 Years) Recurrence of Melanoma: The Scottish Experience,” British Journal of Dermatology 148, no. 2 (2003): 372–373. 10.1046/j.1365-2133.2003.05097_8.x 12588404

